# Strengthening the delivery of asthma and chronic obstructive pulmonary disease care at primary health-care facilities: study design of a cluster randomized controlled trial in Pakistan

**DOI:** 10.3402/gha.v8.28225

**Published:** 2015-11-16

**Authors:** Muhammad Amir Khan, Maqsood Ahmed, Shirin Anil, John Walley

**Affiliations:** 1 Association for Social Development, Islamabad, Pakistan; 2 Public Health Solutions Pakistan, Islamabad, Pakistan; 3 Nuffield Centre for International Health and Development, Leeds Institute of Health Sciences, University of Leeds, Leeds, UK

**Keywords:** asthma, COPD, primary health care, strengthening, enhanced case management

## Abstract

**Background:**

Respiratory diseases, namely asthma and chronic obstructive pulmonary disease (COPD), account for one-fourth of the patients at the primary health-care (PHC) facilities in Pakistan. Standard care practices to manage these diseases are necessary to reduce the morbidity and mortality rate associated with non-communicable diseases in developing countries.

**Objective:**

To develop and measure the effectiveness of operational guidelines and implementation materials, with sound scientific evidence, for expanding lung health care, especially asthma and COPD through PHC facilities already strengthened for tuberculosis (TB) care in Pakistan.

**Design:**

A cluster randomized controlled trial with two arms (intervention and control), with qualitative and costing study components, is being conducted in 34 clusters; 17 clusters per arm (428 asthma and 306 COPD patients), in three districts in Pakistan from October 2014 to December 2016. The intervention consists of enhanced case management of asthma and COPD patients through strengthening of PHC facilities. The main outcomes to be measured are asthma and COPD control among the registered cases at 6 months. Cluster- and individual-level analyses will be done according to intention to treat. Residual confounding will be addressed by multivariable logistic and linear regression models for asthma and COPD control, respectively. The trial is registered with ISRCTN registry (ISRCTN 17409338).

**Conclusions:**

Currently, only about 20% of the estimated prevalent asthma and COPD cases are being identified and reported through the respective PHC network. Lung health care and prevention has not been effectively integrated into the core PHC package, although a very well-functioning TB program exists at the PHC level. Inclusion of these diseases in the already existent TB program is expected to increase detection rates and care for asthma and COPD.

Lung diseases affect people of all age groups and both sexes irrespective of their dwelling. Five major contributors of respiratory diseases, namely chronic obstructive pulmonary disease (COPD), asthma, lung cancer, acute respiratory infections, and tuberculosis (TB), have tremendously increased the morbidity and mortality rates at a global level (1). In 2002, COPD was the fifth leading cause of death, and if the same trend continues, it will become the third leading cause of mortality in 2030 (2). World Health Organization (WHO) has reported that 65 million people suffer from moderate-to-severe COPD, with 90% of COPD deaths occurring in low- and middle-income countries (2). The Global Asthma Report 2014 estimated that 334 million people are suffering from asthma (3) and 80% of asthma deaths occur in low-income and lower-middle-income countries (4).

## Burden of COPD and asthma in Pakistan

Pakistan, a lower-middle-income country, with a population of 182.1 million (5), is facing a high burden of chronic respiratory diseases, including COPD and asthma. Age-standardized mortality rate due to chronic respiratory diseases is estimated to be 138.2 per 100,000 in males and 41.3 per 100,000 among females in Pakistan (6). A large epidemiological survey in the Middle East and North Africa region (BREATHE) conducted in 11 countries reported the prevalence of COPD to be 2.1% among Pakistani adults aged more than 40 years (7). It is expected that the COPD morbidity and mortality rates will increase, in concurrence with the increasing trend of smoking, one of its major risk factors. In Pakistan, prevalence of smoking is 15.2%: 28.6% among men and 3.4% among women (8). Clinical asthma has been estimated to be 4.3% (9), with as high as 10.7% among children aged between 13 and 14 years (10).

## COPD and asthma control in Pakistan

The National Action Plan for prevention and control of non-communicable diseases (NAP–NCDs) was developed in Pakistan in 2003 (11). NAP–NCD highlighted the importance of strengthening the health system in Pakistan to deliver health care for prevention and control of NCDs in the country (12). Despite this, the NCD service delivery model is non-existent. After the Global Alliance against Chronic Respiratory Diseases (GARD) action plan 2008–2013 by the WHO (13), a task force was created and a national coordinator was designated for controlling asthma and allergy conditions. However, progress so far has been suboptimal because of the dissolution of the ministry, lack of funding to GARD, and lack of support from development and technical partners. Spirometry as a standard test for the detection of air flow obstruction is not usually performed in routine care due to which many cases of COPD and asthma remain undiagnosed. A study conducted on 3,696 patients in Karachi showed that 5.7% of the study population had air flow obstruction when spirometry was performed at routine health visits in a primary care setting (14). Another study in Lahore reported that 37.9% of the smokers having ischemic heart disease had undiagnosed COPD (15). Those diagnosed received inadequate treatment because of lack of resources. A cross-sectional study conducted on the availability of essential medicines for the treatment of NCDs showed that inhalers for the treatment of asthma were available only at 0.82% of the 20 public and 60 private outlets surveyed (16).

Pakistan has a health-care infrastructure consisting of basic health units and rural health centers (RHCs) as the first-level care facilities for provision of primary health care (PHC) through the public sector. District headquarter (DHQ) hospitals and tehsil headquarter hospitals are secondary level, and the teaching hospitals in urban areas are the tertiary-level health-care facilities. Though a well-developed public health-care system exists, referral systems are weak or negligible. There is unavailability of diagnostic equipment and lack of specialized training for doctors to detect and treat chronic respiratory diseases (17). Access to the secondary and tertiary hospitals is challenging for those dwelling in the rural areas.

So far, lung health care and prevention has not been effectively integrated into the core PHC package, although a very well-functioning TB program exists at the PHC level. National tuberculosis control program (NTP) in Pakistan has implemented TB control activities in the country with the support of provincial TB control programs in the four provinces. Since 2001, NTP has provided free treatment to 1.5 million TB patients through 5,800 diagnostic and treatment centers that are governed by the public health sector (18).

## Rationale of the study

The proposed research and development project will develop a set of operational guidelines, training packages, and education materials for delivering quality lung health care (especially for asthma and COPD patients) through a district health system built into a functioning TB program. The intervention will then be implemented in selected facilities and evaluated prospectively for effectiveness and feasibility. This randomized controlled trial will address the following gaps in the management of asthma and COPD in Pakistan:

- *Detection rate for asthma and COPD*: Currently, only about 20% of the estimated prevalent asthma and COPD cases are being identified and reported through the respective PHC network (in health management information system reports). Inclusion of these diseases in the already existent TB program is expected to increase detection rates for asthma and COPD. This will be done by improving the diagnostic practices in the RHCs by proper training of doctors through standardized training manuals and ensuring availability of the diagnostic tools and equipment.
- *Prescription practices*: Routine care public health systems offer varied prescription practices by doctors for the management of asthma and COPD. Standardized prescription practices will be implemented in the intervention arm of this trial.
- *Patient education*: There is no systematic patient education available to make patients aware of their chronic respiratory disease in routine care. Patient education tools and behavioral change communication material will be developed and implemented in the RHCs in the intervention arm.
- *Patient follow-up*: The current practice of follow-up is passive, that is, patients approach the health facility as and when required in routine care. This trial will implement the practice of active follow-up, for example, by text messaging the patient for follow-up visits at regular intervals in the intervention arm.
- *Recording and reporting system*: The system for recording and reporting chronic respiratory diseases is inadequate at the RHCs. A standardized recording and reporting system will be implemented in the RHCs for asthma and COPD as in the case of the TB control program.
- *Referral linkages*: As mentioned above, although health-care infrastructure exists in the public health sector in Pakistan, the referral systems are inadequate in routine care. This randomized controlled trial will develop and apply a proper referral linkage system in the intervention arm.
- *Monitoring and evaluation*: At present, monitoring and evaluation (M&E) for detection and management of asthma and COPD is inadequate. M&E system will be developed and executed through this trial to provide quality care for asthma and COPD patients in Pakistan.

## Objectives

The primary objective of this study is to establish the clusters successfully and thereafter compare the control of asthma and COPD in the patients receiving quality lung health care in the intervention clusters with those receiving routine care in PHC setting in the control arm clusters in Pakistan.

Secondary objectives are:

- to achieve 50% or more quit rates among cigarette-smoking asthma and COPD patients;
- to understand and document the care delivery experiences of patients, care providers, and managers;
- to conduct incremental cost-effectiveness analysis of managing asthma and COPD at PHC facilities in Pakistan; and
- to inform the country plans for scaling-up quality lung health care in Pakistan and elsewhere.

## Methods

### Study design, duration, and setting

**Fig. 1:**
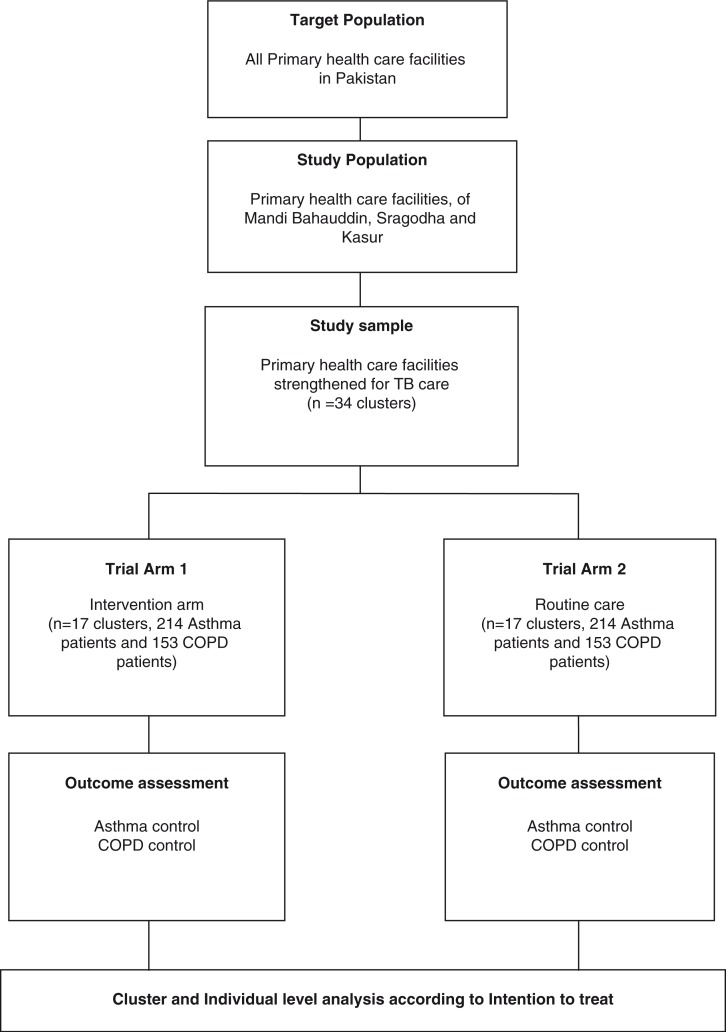
Design of the trial. COPD, chronic obstructive pulmonary disease; TB, tuberculosis.

This cluster randomized controlled trial with two arms is being conducted from October 2014 to December 2016. Intervention will be provided in Arm 1 as compared with the routine care in Arm 2 (Fig. 1). The districts of Mandi Bahauddin, Sargodha, and Kasur in Pakistan have been selected for the proposed research.

### Study population

Thirty-four RHCs and subdistrict hospitals, already strengthened for TB care, will be recruited in the study. An RHC and subdistrict hospital is a facility that serves a population of 160,000 or more. The facility staff includes a) three or more doctors, b) a range of paramedics including lady health visitors, nurses, dispensers, laboratory technicians, and c) support staff such as record clerks, store keepers, cleaners, and so on. The facility offers diagnostic and treatment services for communicable diseases (TB, malaria, acute respiratory infection, diarrhea, etc.); basic emergency obstetric and neonatal care; and NCDs such as malnutrition, hypertension, diabetes mellitus, asthma, and COPD. The currently available lung health services include a) clinical services – clinical consultation and drugs and b) support services – investigation, education, and so on.

**Fig. 2:**
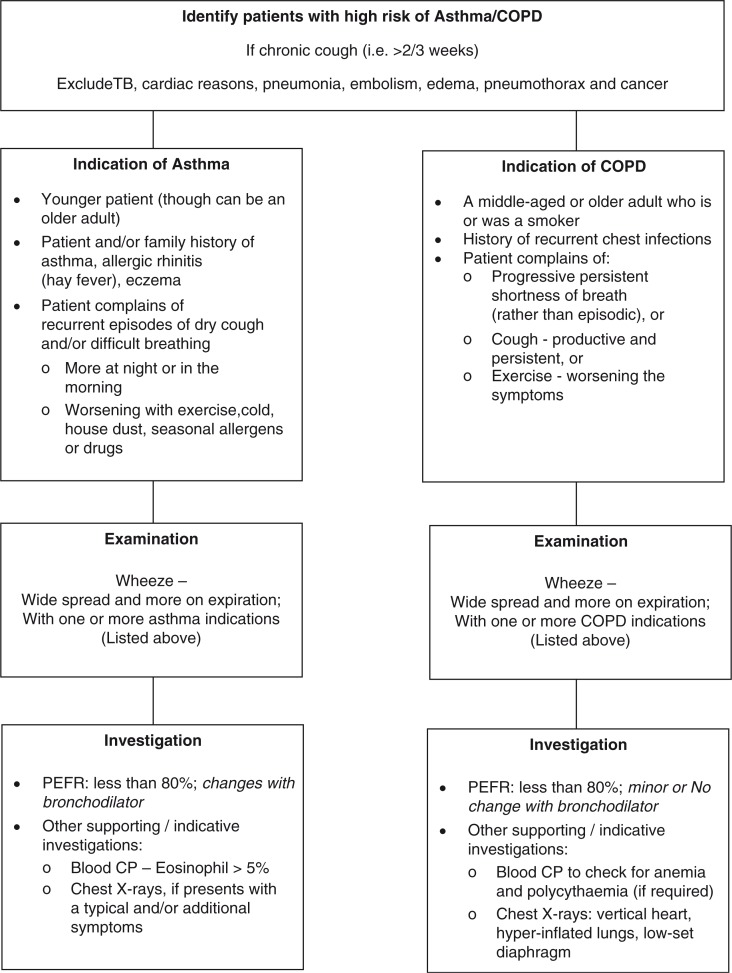
Diagnosis of asthma and chronic obstructive pulmonary disease (COPD). PEFR, peak expiratory flow rate; TB, tuberculosis.

All newly diagnosed asthma/COPD patients, elaborated in Fig. 2, based on the guidelines of Global Initiative for Chronic Obstructive Lung Diseases (19), Global Initiative for Asthma (20), National Institute for Health and Clinical Excellence (NICE) (21, 22), and WHO (23), aged 18 years or more, and currently residing (and expected to continue residing for the next 12 months) in the catchment area of the respective health facility (i.e. RHC or subdistrict hospital), are eligible for inclusion in the trial. The reason for including asthma and COPD patients aged more than 18 years is that airflow obstruction was found even at a young age in primary care settings in Pakistan (14); COPD was also found in patients aged less than 40 years in tertiary care hospitals in Karachi (24). Patients fulfilling the inclusion criteria, but who refuse to be a part of the trial, will be excluded.

### Intervention

#### Intervention arm

PHC facilities in the intervention arm will be strengthened to diagnose and manage asthma and COPD patients. Strengthening of PHC facilities for case management enhancement mainly includes availability of context-sensitive guidelines and materials for case management; health staff trained on operational guidelines and materials; supplement material support for managing asthma and COPD including patient education tools for asthma and COPD awareness and smoking cessation; standardized recording and reporting; enhanced facility monitoring; facilitated referral linkages with DHQ hospital; and better retrieval of patients with delayed follow-up visits.

#### Control arm

The control for comparison is ‘routine care’ for asthma and COPD case management at PHC facilities. The only addition will be introduction of standard diagnosis and recording practices for asthma/COPD patients attending these control facilities and continued provision of essential drugs (at the current level) through the district health office.

### Outcome assessment

For asthma, trained facility staff, at the start and then after 6 months of treatment, will: (a) assess five clinical indicators (waking up at night, use of β-2 agonist, restriction of daytime activity, emergency/urgent treatment for asthma and missing work/ school due to asthma)–using a standardized questionnaire as used by other researchers (25, 26), absence of which indicates asthma control and decreased rate of exacerbations; and (b) conduct spirometry – using digital spirometer: Forced expiratory volume in 1 (FEV1), Forced vital capacity (FVC) and FEV1/FVC ratio will be measured. Asthma treatment approach includes the use of ‘Reliever’ for quick relief of symptoms such as the use of salbutamol inhaler 100 µg/puff at a dose of two puffs twice or thrice daily up to a maximum of two puffs four times a day, use of ‘Controller’ for regular symptom control like beclomethasone inhaler 250 µg/puff at a dose of two puffs twice a day up to a maximum of two puffs four times a day, or long-acting β-2 agonist salmeterol inhaler 250 µg/puff at a dose of two puffs twice a day up to a maximum of four puffs twice a day; if symptoms are not controlled, for example, occur >3 times a week, step-up treatment will be considered, and if control is maintained for ≥3 months, step-down regimen will be considered.

Control of COPD will be monitored by trained facility staff by measuring BODE (Body mass index, airway Obstruction, Dyspnea and Exercise capacity) index (27, 28) before and after 6 months of the intervention. COPD treatment will consist of salbutamol 100 µg/puff at a dose of two puffs three or four times daily, ipratropium 20 µg/puff at a dose of two puffs two to four times daily, or salbutamol and ipratropium, if symptoms persist, for more than 2 weeks on individual regimen in mild cases; salmeterol 250 µg/puff and fluticasone 25 µg/puff at a dose of two puffs two times daily up to a maximum of four puffs twice daily in moderate-to-severe cases; if symptoms are not adequately reduced in 2 weeks, long-acting muscarine antagonist, for example, tiotropium 18 µg capsule, would be added to the regimen once daily, or sustained release of oral theophylline 200 mg tablet would be administered twice daily.

### Randomization

Eligible clusters (health-care facilities) will be randomized into the two trial arms by using the lottery method, which will be executed at the central trial unit at Association for Social Development, monitored by the Trial Steering Committee.

### Blinding

Because of the nature of the trial, blinding of the individual health-care providers and patients will not be possible. Nevertheless, the data analyst will be blinded to the intervention and the control arms.

### Patient follow-up

Registration of the patients for the trial will continue for 9 months. All the patients included in the study will be followed up for 6 months, visiting the health-care facility on a monthly basis for medical consultation and drug administration.

### Data collection

Data will be collected through trial registers at the included health-care facilities by trained data collectors, supervised by the field manager and monitored by the project manager. Data inconsistency and missing data will be handled by verifying with the trial register and contacting the patient/doctor/facility staff.

### Costing study

A costing study will be designed and conducted with a focus on health-care- and patient-related cost. Cost-effectiveness analysis (cost-to-effect ratio) will be done to compare the incremental costs related to asthma and COPD management in both the intervention and control arms with respect to the outcomes achieved.

### Exploratory study

Key informant interviews and focused group discussions will be done with district managers (*n*=3), care providers (*n*=8), and patients (*n*=16). This will help in explaining the results of the study and inform the researchers and the policymakers about the social impact of the intervention for asthma and COPD control at the district level.

### Statistical considerations

#### Sample size

For asthma intervention – at least with 17 clusters with 428 asthma patients would be needed to detect an absolute difference of 20% control of asthma between intervention and control arm at six months with 95% confidence and 80% power, considering attrition rate of 20% and intracluster correlation coefficient (ICC) of 0.05. For COPD intervention – at least 17 clusters with 306 COPD patients will be required to detect a mean score change in BODE index of at least 1.0, taking standard deviation of BODE index as 2.0, with 95% confidence and 80% power, ICC of 0.05 and assuming attrition rate of 20%.

#### Analysis

Individual- and cluster-level analyses will be done according to intention to treat. Baseline characteristics will be compared between the intervention arms to see if randomization resulted in equal distribution of characteristics, the purpose of randomization. Change in the control of asthma and BODE index for COPD control will be measured in each arm and the difference between the arms will be measured by applying the specific statistical tests at 6 months. Residual confounding will be taken care of by running logistic regression model for control of asthma and linear regression model for COPD control measured by BODE index, adjusting for clustering in the models. A *p*-value of less than 0.05 will be considered significant in all the analyses.

### Ethical considerations

The study will be conducted in accordance with the ethical principles as laid down in the declaration of Helsinki. Informed consent will be obtained from all the participants of the trial and their confidentiality and privacy will be adhered to at all times. The study has been ethically approved by the National Bioethics Committee of Pakistan (Ref. No. 4-87/NBC-111/13/RDC/4682) and the ethical research committee at the University of Leeds (Ref. No. HSLTM/12/025).

## Expected outcome

This trial will provide standardized guidelines and tools such as a doctor's training manual and desk guide for the management of asthma and COPD at the PHC level; patient education material for asthma and COPD awareness and smoking cessation; and recording, reporting, and referral systems for diagnosis and management of asthma and COPD patients in the public health sector of Pakistan. NICE guidelines for COPD, introduced in 2004, had a positive impact on the diagnosis and management of COPD patients (29). Pont et al. (30) have reported that asthma patients in whom standard guidelines were followed had better health-related quality of life as compared with those receiving non-guideline treatments. Hence, it is expected that this trial will not only lead to better asthma and COPD control in the intervention arm as compared with the control arm (routine care) but also to a better quality of life in patients in the intervention arm. It is also expected that smoking quit rate will be more in the intervention arm as compared with the control, as found in a study by Hilberink et al. (31), in which smoking cessation counseling with intensified minimal intervention strategy was provided to COPD patients in general practice, as is being provided in the intervention arm of this trial.
